# Roles of resonant muonic molecule in new kinetics model and muon catalyzed fusion in compressed gas

**DOI:** 10.1038/s41598-022-09487-0

**Published:** 2022-04-16

**Authors:** Takuma Yamashita, Yasushi Kino, Kenichi Okutsu, Shinji Okada, Motoyasu Sato

**Affiliations:** 1 Institute for Excellence in Higher Education, Tohoku University, Sendai, Miyagi 980-8576 Japan; 2 Department of Chemistry, Tohoku University, Sendai, Miyagi 980-8578 Japan; 3 Engineering Science Laboratory, Chubu University, Kasugai, Aichi 487-8501 Japan

**Keywords:** Physics, Atomic and molecular physics, Nuclear physics, Quantum physics, Engineering, Energy infrastructure

## Abstract

Muon catalyzed fusion ($\mu$CF) in which an elementary particle, muon, facilitates the nuclear fusion between the hydrogen isotopes has been investigated in a long history. In contrast to the rich theoretical and experimental information on the $\mu$CF in cold targets, there is relatively scarce information on the high temperature gas targets of deuterium-tritium mixture with high-thermal efficiency. We demonstrate new kinetics model of $\mu$CF including three roles of resonant muonic molecules, (i) changing isotopic population, (ii) producing epi-thermal muonic atoms, and (iii) inducing fusion in-flight. The new kinetics model reproduces experimental observations, showing higher cycle rate as the temperature increasing, over a wide range of target temperatures ($T<800$ K) and tritium concentrations. Moreover, it can be tested by measurements of radiative dissociation X-rays around 2 keV. High energy-resolution X-ray detectors and intense muon beam which are recently available are suitable to reveal these dynamical mechanism of $\mu$CF cycles. Towards the future $\mu$CF experiments in the high-temperature gas target we have clarified the relationship between the fusion yield and density-temperature curve of adiabatic/shock-wave compression.

## Introduction

Nuclear fusion reactors have been pursued for a long time with prospect of future energy source^1^. In general, confinement of hydrogen isotope plasma is necessary for fusion, and the major challenge of the reactor development is to create and maintain plasma of several 10$^8$ K by magnetic or inertial confinement^2,3^. Another confinement mechanism is known as “chemical confinement” by an elementary particle muon ($\mu$)^4^. Because of the 207 times larger mass of $\mu$ than an electron, the $\mu$ can strongly squeeze the two hydrogen nuclei and form a muonic molecule in which the nuclear fusion reaction occurs by the overlap of nuclear wave function. The idea of an intramolecular fusion (IMF) was proposed by Frank and Sakharov independently^5,6^, followed by more detailed theoretical considerations by Zeldovich^7^. Experimental observation of IMF was first reported in 1956^8^, where a muonic molecule $\mathrm {pd}\mu$, in which $\mu$ binds itself by a proton p and a deuteron d with the binding energy 220 eV, forms in a hydrogen bubble chamber and the fusion reaction of $\mathrm {pd}\mu \rightarrow \,^3\mathrm {He}+\mu$ was recorded. The other fusion events in a deuterium chamber, $\mathrm {dd}\mu \rightarrow \,\mathrm {t}+\mathrm {p}+\mu$, were also recorded in 1963^9^. Here, t is a triton. Since the $\mu$ itself does not directly enter the nuclear reaction but acts like a catalyst for chemical reactions, these phenomena were reported as “Catalysis of nuclear reactions”.

The idea of muon catalysed fusion ($\mu$CF), therefore, stems from the repeated reactions of IMF. In the early stage of the $\mu$CF history, the number of $\mu$CF event per $\mu$ was considered to be too low to be used as a fusion reactor. The experiments by Dzhelepov et al.^10^, however, showed a much higher molecular formation rate of dd$\mu$ than the theoretical prediction assuming the mechanism in which the binding energy of dd$\mu$ transfers to an electron in the collision between d$\mu$ and D$_2$. Based on the above experimental observation and theoretical suggestions^11^, Vesman proposed another mechanism of dd$\mu$ formation in 1967^12^, where the formation of muonic molecules occurs resonantly transferring its binding energy to the rovibrational excitation of target D$_2$ molecules, e.g.,

1 $$\begin{aligned} \mathrm {d}\mu (1s) + \mathrm {D}_2(J_i,\upsilon _i) \rightarrow [(\mathrm {dd}\mu )\mathrm {dee}](J_f,\upsilon _f), \end{aligned}$$

where $J_{i/f},\upsilon _{i/f}$ are rotational and vibrational quantum numbers of the initial/final states. This so called Vesman mechanism (VM) results in a temperature dependency of muonic molecular formation rates, and was confirmed experimentally for dd$\mu$^13^. The same idea was deduced to the muonic molecule dt$\mu$ in which the d and triton t are confined by $\mu$, and was confirmed by experiments^14^. Theoretical studies based on few-body quantum mechanics had established the existence of rovibrationally-excited bound states of dd$\mu$ and dt$\mu$ whose binding energies are 1.97 and 0.66 eV, respectively. These loosely bound states are compatible with the host-molecular rovibrational excitation, $(J_i,\upsilon _i)\rightarrow (J_f,\upsilon _f)$, as Vesman proposed.

Owing to the non-zero amplitude of the wave function of the d-t motion at the origin inside the compact size of the muonic molecules, an intramolecular fusion (IMF) occurs immediately at the rate of $10^{12}$ s$^{-1}$ for dt$\mu$, $10^{8}$ s$^{-1}$ for $\mathrm {dd}\mu$, and $10^{7}$ s$^{-1}$ for $\mathrm {tt}\mu$. For dt$\mu$, the IMF,

2 $$\begin{aligned} \mathrm {dt}\mu \rightarrow \alpha + \mathrm {n} + \mu + 17.6\,\mathrm {MeV}, \end{aligned}$$

results in a “liberalized” $\mu$. The released $\mu$ repeatedly undergoes the muonic molecular formation and subsequent fusion; the circular process including VM and IMF is called $\mu$CF cycle. The $\mu$CF cycle has been investigated dedicatedly with the expectation of future energy/neutron sources^4,15–18^. The $\mu$CF kinetics model based on the VM is now well established in cold D$_2$ and H$_2$/D$_2$ systems^19,20^.

With respect to the application of $\mu$CF to energy sources, the cold target of D$_2$ and H$_2$/D$_2$ is unrealistic owing to its low thermal efficiency. The higher thermal efficiency of $\mu$CF can be achieved in a hot target. In addition to the thermal efficiency, the much higher fusion yield can be achieved in D$_2$/T$_2$ mixtures because of the highest formation rate among the muonic molecule isotopologues, rapid fusion rate, and the smallest probability of released $\mu$ to stick $\alpha$. Despite of the highest efficiency, however, the experimental data of $\mu$CF in D$_2$/T$_2$ targets are relatively scarce, partly due to the difficulty of T$_2$ handling. Kawamura et al. reported the anomalous temperature dependency of the $\mu$CF cycle rate^21^ using a solid D$_2$/T$_2$ target in the temperature range of 5–16 K. Bom et al. summarized the $\mu$CF experimental results obtained at the Joint Institute for Nuclear Research phasotron (JINR)^22^ since 1997. The experimental conditions covered wide range of target temperatures of 20–800 K, densities of 0.2–1.2 of the liquid hydrogen density (LHD), and tritium concentrations of 15–86%. They observed increments of the cycle rate as both temperature and density. They pointed out the importance of $\mu$CF experiments at high temperatures above 1000 K.

In a kinetics model of $\mu$CF, the number of fusion events catalyzed by one $\mu$, $Y_\mathrm {f}$, can be related with the cycle rate $\lambda _c$ and a $\alpha \mu$ sticking probability *W* as

3 $$\begin{aligned} Y_\mathrm {f}^{-1}=W+\lambda _0/\lambda _c\varphi , \end{aligned}$$

where $\lambda _0=0.455\times 10^6$ s$^{-1}$ is the $\mu$ decay rate^23^ and $\varphi$ is the number density of target hydrogen atoms relative to the liquid hydrogen density (LHD; $4.25\times 10^{22}$ atoms cm$^{-3}$). The parameter *W* represents the muon loss probability from the cycle, namely the $\alpha \mu$ sticking that the muon captured the atomic orbital of the $\alpha$ just after the fusion reaction. Although the theoretical representation of *W* depends on the kinetics model under consideration, in a simple model based on the dt$\mu$ fusion cycle, *W* is often given by $W=(1-R)\omega _s$ where $\omega _s\simeq 0.8\%$ is an initial sticking probability of $\mathrm {dt}\mu \rightarrow \alpha \mu + \mathrm {n}$^15^ and $R\sim 0.35$ represents reactivation fraction^24^, i.e. muon stripping probability from $\alpha \mu$ in collisions with surrounding D$_2$/T$_2$. Using typical values of $W=0.5$% and $\lambda _c\sim 10^8$ s$^{-1}$ results in $Y_\mathrm {f}\sim 100$ at LHD.

The cycle rate can be approximated as

4 $$\begin{aligned} \frac{1}{\lambda _c} \approx \frac{1}{\lambda _{\mathrm {dt}\mu } c_\mathrm {d}} + \frac{c_\mathrm {d}q_{1s}}{\lambda _\mathrm {dt}c_\mathrm {t}}, \end{aligned}$$

where $c_\mathrm {d}$ and $c_\mathrm {t}$ are fraction of D$_2$ and T$_2$, respectively, and satisfy $c_\mathrm {d}+c_\mathrm {t}=1$. $\lambda _{\mathrm {dt}\mu }$ is the dt$\mu$ formation rate via the VM, $\lambda _\mathrm {dt}\simeq 10^{8}$ is a muon transfer rate between the ground state muonic atoms, d$\mu (1s)$ + t $\rightarrow$ d + t$\mu (1s)$. The $0\le q_{1s}\le 1$ is a phenomenological factor (deduced from experiments) that represents the probability of a d$\mu ^*$ reaching d$\mu (1s)$. The Eq. (4) manifests the importance of VM in that the $\lambda _{\mathrm {dt}\mu }$ plays a primary role to realize high $\lambda _c$.

In the higher temperature region of 100–800 K, several experimental data^15,22,25^ clearly indicate higher $\lambda _c$ and its increase as temperature. This amplification of $\lambda _c$ at high temperatures could be caused by an increase in the $\mathrm {dt}\mu$ formation rate $\lambda _{\mathrm {dt}\mu }$. A beam experiment^26^ utilizing the Ramsauer-Townsend effect of muonic atoms revealed a significantly high $\lambda _{\mathrm {dt}\mu } = (7.1\pm 1.8)\times 10^9$ s$^{-1}$ at the resonance energy 0.423±0.036 eV for the reaction $\mathrm {t}\mu$ + D$_2$ $\rightarrow$ $[\mathrm {dt}\mu \mathrm {dee}]_{\upsilon =3}$. This value is much higher than the typical formation rate $\lambda _{\mathrm {dt}\mu }\sim 10^8$ s$^{-1}$ at lower energy conditions, and even higher than perturbative calculations, including quadrupole correction under full-thermalization conditions^27^.

In these two decades, theoretical investigations associated with experiments including time-of-flight and X-ray spectroscopy have shed a light on the further understanding of muonic atom processes. These studies were motivated by solid state effects on VM^28,29^, Ramsauer-Townsend effect on muonic atom scattering^30^ $\mu$CF target optimization^31^, and refinement of cascade models^32–35^ that are related to the precise measurement of muonic hydrogen energy levels^36–42^.

In addition to these progresses, the $\mu$CF is recently rejuvenated with new sophisticated techniques and renewed motivation. The improvement of the energy resolution of X-ray detectors allows us to reveal the dynamics of muon atomic processes^43–45^ in more detail. Intense muon beam^46^ also provides upgraded conditions for such experiments. As well as the energy source, a $\mu$CF-based neutron source is another motivation. Since the neutron emitted from the d-t fusion has mono-energetic spectrum, it can be used to reduce long-lived fission products (LLFPs), or the high-level radioactive waste from nuclear power plants; the idea itself of transmutation of LLFPs by a $\mu$CF-based neutron beam was described in literature^47–49^ whereas it has not been featured in these decades. A new concept of gas target composed of conical spatially localized D/T mixture gas streams for $\mu$CF combined with resonance rf acceleration techniques was reported recently^50^. The released $\mu$ after the fusion would have 10 keV kinetic energy on average and would be utilized by muon beam cooling^51^ which can be applicable to negative muon microscope and injection source of muon collider^52^ exploring beyond standard model of particle physics. The exact energy distribution of the released muon after the fusion was recently calculated^53^, while experiments for observation of these muons using the high-intensity pulsed muon beam are in progress^54–56^.

The aim of this study is to explore the possibility of $\mu$CF at the higher temperature gas target ($T<10^4$ K and $10^{-3}<\varphi <1$). For this purpose, we propose a new kinetics model including resonant muonic molecules that play several crucial roles in the $\mu$CF cycles. We have solved coupled rate equations based on the new kinetics model by the 4th-order Runge-Kutta method and investigated the responses of $Y_\mathrm {f}$ and $\lambda _c$ to the uncertainty of the rate parameters. We present the theoretical model shows a fairly good agreement with experimental observations. Toward the advent of a hydrogen-based society, it is becoming possible to handle high-temperature, high-density hydrogen safely and at low cost. A high-temperature and high-pressure hydrogen gas target by shock wave, which is advantageous for extracting energy and neutrons by $\mu$CF, has been proposed^57^. We demonstrate the new $\mu$CF kinetic model in the new gas target condition.

## Options of the mechanism

The kinetic energy distribution of the muonic atoms has caused arguments in which it will change the effective rate of the $\mathrm {dt}\mu$ formation. An electron in the molecule is first replaced by an injected $\mu$ and a highly excited muonic atom ($n\sim 14$) is formed. Subsequently, the muonic atom cascades down to lower levels, where part of the level transition energy converts to its kinetic energy. In addition, an isotopic muon transfer reaction

5 $$\begin{aligned} \mathrm {d}\mu (n) + \mathrm {t} \rightarrow \mathrm {t}\mu (n) + \mathrm {d} + 48/n^2 \mathrm {eV}, \end{aligned}$$

where *n* denotes the principal quantum number of the muonic atoms, produces a $\mathrm {t}\mu$ atom with epi-thermal energy. The muonic atom cascade processes have been investigated experimentally from X-ray measurements of K$_\alpha$/K$_\beta$ ratio^58–61^. Recently, close-coupling calculations were performed for the Coulomb deexcitation in p$\mu$-H and d$\mu$-D collisions^32–35^, in which the muonic atoms are accelerated by the $n\rightarrow n'$ ($n>n'$) deexcitation energy in addition to the isotopic muon transfer. Owing to these collisional processes of excited muonic atoms, it is rational to consider that some muonic atoms that reach ground states have epi-thermal kinetic energies and, consequently, the molecular formation rate via VM deviates from the full-thermalization condition^62,63^. So far the epi-thermal effects have been investigated by Monte-Carlo simulation^64–70^ and experiments using low-density gas targets^71^. The epi-thermal effects on the steady state of $\mu$CF at high density ($\varphi \ge 0.4$) were examined by analyzing the experimental data^72^. At any target conditions, the comparison between the theory and experiments suggested amplification of the dt$\mu$ formation rate $\lambda _{\mathrm {dt}\mu }$ by the epi-thermal effects. However, the time dependence of the kinetic energy distribution of muonic atoms has still involved unknown factors and the correspondence between the theory and experiments has not completed yet.

**Figure 1:**
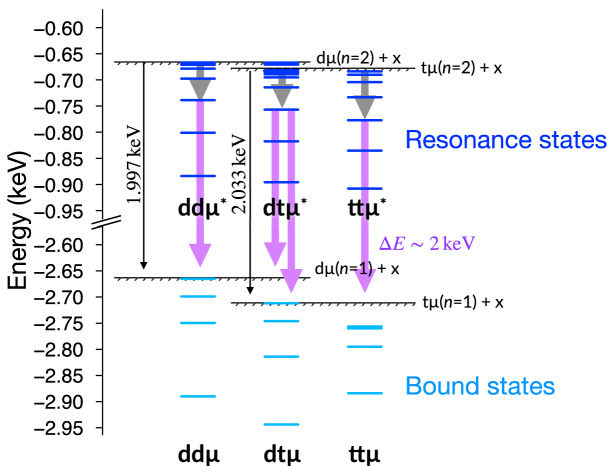
Energy level diagram of resonance states (restricted to rotationally ground state and its vibrational excited states ) and bound states (all rovibrational states) of muonic molecules. The hatched areas indicate the VM range 5 eV below the threshold energy. x denotes d or t.

In addition to the VM that plays an important role in the formation of bound muonic molecules, a side-path model (SPM)^73–75^ has been proposed where the formation of resonant muonic molecules $$\mathrm {dt}\mu ^*$$, $$\mathrm {dd}\mu ^*$$, and $$\mathrm {tt}\mu ^*$$^40,76–79^ is included. These resonant muonic molecules are expected to form by the same process as the VM in which the excess energy of formation transfers to the rovibrational excitation of D$$_2$$/T$$_2$$, e.g.,

6 $$\begin{aligned} \mathrm {t}\mu (n=2) + \mathrm {D}_2(J_i,\upsilon _i) \rightarrow [(\mathrm {dt}\mu ^*)\mathrm {dee}](J_f,\upsilon _f), \end{aligned}$$

where $J_{i/f},\upsilon _{i/f}$ are rotational and vibrational quantum numbers of the initial/final states. Owing to the degeneracy of the $n=2$ energy levels in the muonic atoms, the resonant muonic molecules have several rovibrational energy levels produced by the long range induced dipole potential below the $\mathrm {d}\mu$/$\mathrm {t}\mu$($n=2$) + D$_2$/T$_2$ threshold energy, which leads to much higher rates of molecular formation around $\lambda _\mathrm {SPM}\simeq 10^{11}$ s$^{-1}$ than $\lambda _{\mathrm {dt}\mu }\simeq 10^{8}$ s$^{-1}$. Figure 1 illustrates the energy level diagram of muonic molecules based on the few-body quantum mechanical calculations (see review articles for the bound states^4,15^ and above mentioned references for the resonances). The $\mathrm {d}\mu$/$\mathrm {t}\mu$($n=2$) + x (x denotes d or t) threshold energy is located approximately 2 keV above the lowest threshold energy of $n=1$. The resonance energy levels are accumulated to the $\mathrm {d}\mu$/$\mathrm {t}\mu$($n=2$) + x threshold energy. As indicated by the shaded region, the VM-like mechanism requires the energy levels located below the threshold energy by $\lesssim 5$ eV so that the excess energy of the molecular formation is compatible with the host-molecular rovibrational excitation. Thus, the resonance states would form in vibrationally excited states and then undergoes deexcitation processes where the excess energy is shared by the dissociation fragments or emitted as a photon. The contribution of SPM to the kinetics model of $\mu$CF was reported in Ref.^73^ where the dissociation of $\mathrm {dt}\mu ^*$ changes the population of $\mathrm {d}\mu$ and $\mathrm {t}\mu$ from the VM. The SPM explains the $\lambda _c$ in a wide range of tritium concentrations $0\le c_\mathrm {t}\le 1$; however, the contribution of other resonant muonic molecules, dd$\mu ^*$ and tt$\mu ^*$, and the application of SPM to the high-temperature region have not been discussed in detail thus far.

Precise three-body variational calculations indicate that branching ratios of the radiative dissociation of the dt$\mu ^*$ and dd$\mu ^*$ are 0.9^79–82^. The X-ray emitted from the radiative dissociation has a peak close to 2 keV, suggesting that the dissociated muonic atoms would have a few tens of eV. Therefore, the prediction of the kinetic energy distribution of muonic atoms becomes more challenging. The non-radiative dissociation produces a ‘hot’ muonic atom with kinetic energy of approximately 1 keV because the muonic atom shares half of the entire dissociation energy 2 keV with the other similar mas fragment.

Another process is fusion in-flight (FIF)^83^, in which the collision between $\mathrm {t}\mu$ (1*s*) and $\mathrm {d}$ leads to the nuclear fusion without muonic molecular formation. As the $\mu$ strongly screens the Coulomb repulsion between $\mathrm {d}$ and $\mathrm {t}$, the collision energy required for FIF is much lesser than that for the d-t bare nuclear collisions. The fusion rates of FIF were reported for collision energies *E* up to 10 keV, where significant non-adiabatic effect was predicted^83^. Under non-ionized target gas conditions ($<10^4$ K), the contribution of FIF to the fusion cycle should be negligible except for the ‘hot’ muonic atoms considered in this paper. So far, the contribution of the FIF associated with the SPM to the $\mu$CF kinetics model has never been considered so far.

## Kinetics model

We propose a new kinetics model including the VM, SPM, and FIF, with particular focus on the three roles of the resonant muonic molecule, namely, (i) dt$\mu ^*$ changes isotopic population of d$\mu$ and t$\mu$, (ii) *all* species of the resonant muonic molecules produce epi-thermal muonic atoms, and (iii) the ‘hot’ muonic atoms induce fusion in-flight. In order to consider the epi-thermal effect of the muonic molecules in the VM, we introduce a simple scaling factor $1\le \eta _{\mathrm {dt}\mu }$ and define the temperature-dependent $\mathrm {dt}\mu$ formation rate $\lambda _{\mathrm {dt}\mu }(T)$ as $\lambda _{\mathrm {dt}\mu }(T)=\eta _{\mathrm {dt}\mu }\lambda _{\mathrm {dt}\mu }^{(\mathrm {theo})}(T)$, where $\lambda _{\mathrm {dt}\mu }^{(\mathrm {theo})}(T)$ is given theoretically by Faifman et al.^27^ under the full-thermalization condition. The $\lambda _{\mathrm {dt}\mu }^{(\mathrm {theo})}(T)$ is defined independently for the spin state ($F=0,1$) of $\mathrm {t}\mu$ (1*s*). Hereinafter, we refer to the VM enhanced by $\eta _{\mathrm {dt}\mu }$ as an enhanced-VM (EVM). The main purpose of the present work is to compile the major frameworks of $\mu$CF mechanism and to overview the fusion and X-ray yields as a function of temperature and target densities towards future applications and recent precise X-ray spectroscopy. Therefore, we present calculations based on the coupled rate equations which are advantageous to deal with the sequential reactions and obtain the integrated yields of various signals within small computational cost. Although the present calculations are disadvantageous to treat thermalization of the atoms, the epi-thermal effect can be approximately incorporated to the formation rate with scaling factor.

The formation rate of the resonant muonic molecules $\lambda _\mathrm {SPM}$, involves ambiguity and is tuned to reproduce the experimental results in the range of $10^{10}\,\mathrm {s}^{-1}\le \lambda _\mathrm {SPM}\le 10^{12}\,\mathrm {s}^{-1}$ that covers the rates used in Ref.^73^ under the full-thermalization condition and experimental suggestions^40^.

The fusion in-flight rate $\lambda _\mathrm {FIF}(E)$ was calculated as a function of the collision energy *E*^83^. Although the calculated fusion in-flight rate $\lambda _\mathrm {FIF}(E)$ unphysically oscillate against the collision energy, we obtain smooth function after averaging with the Boltzmann distribution.

**Figure 2:**
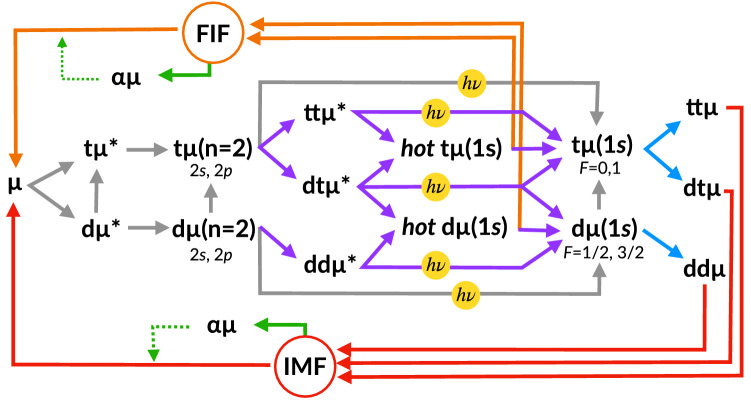
Reaction scheme of $\mu$CF, including the $\mathrm {dt}\mu$ formation based on the Vesman mechanism (VM, lightblue arrows) and subsequent intramolecular fusion (IMF, red arrows), side-path model (SPM, purple arrows), and fusion in-flight (FIF, orange arrows). The green arrows denote the $\alpha \mu$ sticking and the dashed green arrow denotes $\mu$ reactivation from $\mu$He. The arrows with $h\nu$ indicate X-ray emissions from the 2*p* state muonic atoms and resonance states of the muonic molecules.

Figure 2 summarizes the EVM-SPM-FIF model of $\mu$CF considered in this work. Some of t$\mu$ ($n=2$) and d$\mu$ ($n=2$) are subject to the SPM, and the others are deexcited to the ground state. The hot muonic atoms produced by the dissociation of resonant muonic molecules undergo FIF, which competes with thermalization. The IMF and FIF subsequently occur and the $\mu$ becomes free again. Some of the $\mu$ stick to the helium nucleus after fusion and in part, depending on the temperature, are reactivated in collisions with target molecules^24^.

We solve the kinetics model using the 4th-order Runge-Kutta method with a time step $\Delta t <10^{-14}$ s. The atom/molecule ratio $f_\mathrm {mol}(T),f_\mathrm {at}(T)$ of the target gas is estimated by the law of mass action using the binding energy of the D$_2$/T$_2$ molecules. These fractions are considered in the VM and SPM rates.

The other rate constants, such as the $\mu$ capture rate, cascade down rates, Stark mixing rate between 2*s* and 2*p* of d$\mu$/t$\mu$, spin-flip collisions (depending on the temperature), dd$\mu$ and tt$\mu$ formation rates, and reactivation probability (depending on temperature) are set to the previously reported values^15,20,24,84^.

The number of fusion events is calculated by

7 $$\begin{aligned} \frac{\mathrm {d}Y_\mathrm {f}}{\mathrm {d}t}&= \lambda _{\mathrm {f}}^{(\mathrm {dt})}N_{\mathrm {dt}\mu }(t) + \lambda _{\mathrm {f}}^{(\mathrm {dd})}N_{\mathrm {dd}\mu }(t) + \lambda _{\mathrm {f}}^{(\mathrm {tt})}N_{\mathrm {tt}\mu }(t) + \lambda _\mathrm {FIF} \left( c_\mathrm {t}\varphi N_{\mathrm {d}\mu (\mathrm {hot})} + c_\mathrm {d} \varphi N_{\mathrm {t}\mu (\mathrm {hot})}\right) , \end{aligned}$$

where $N_{i}(t)$ represents the population of *i*. We use $\lambda _\mathrm {FIF}=2\times 10^8$ s$^{-1}$ which corresponds to the hot muonic atom collision in $\sim 1$ keV kinetic energy produced from the non-radiative dissociation of resonance states of muonic molecules.

## Reproducibility of experimental observations

**Figure 3:**
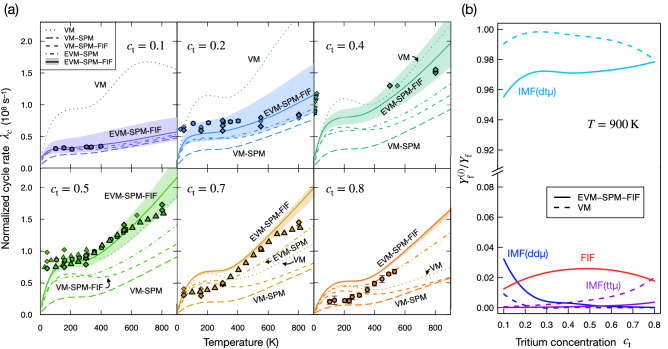
(**a**) Normalized cycle rates $\lambda _c$ calculated at $\varphi =0.4$ are shown as a function of target temperature. The lines are calculation and symbols are experimental results: $\circ$^25^, $\triangle$^15^, $\triangledown$^21^, $\diamond$^22^. The bold symbols denote the values obtained in $0.3\le \varphi \le 0.5$ conditions. The shade range is drawn in the vicinity of $0.1\le \Upsilon _{\mathrm {t}\mu }\le 0.9$ (the solid line corresponds to $\Upsilon _{\mathrm {t}\mu }=0.5$; dt$\mu ^*$ is assumed to result in t$\mu$ and d$\mu$ equally). Note that the data points of $c_\mathrm {t}=0.4$ and $T<16$ K are from experiments using a solid D$_2$/T$_2$ target^21^. (**b**) Contribution of IMF and FIF to the total fusion yield as a ratio of fusion yield $Y_\mathrm {f}^{(i)}/Y_\mathrm {f}$ where $Y_\mathrm {f}$ denotes total fusion yield and $Y_\mathrm {f}^{(i)}$ denotes partial fusion yields whose component *i* is noted by the lines. The conditions are $\Upsilon _{\mathrm {t}\mu }=0.5$, $\varphi =0.4$ and $T=900$ K.

Figure 3a displays the calculated cycle rates $\lambda _c$ together with the available experimental results in wide temperature ranges. We display the results of EVM-SPM-FIF kinetics model, where $\eta _{\mathrm {dt}\mu }=5$ and $\lambda _{\mathrm {SPM}}=5\times 10^{10}$ s$^{-1}$, and compare the experimental data with other models with different parameters. As the branching ratio resulting in $\mathrm {t}\mu (1s)$ after the radiative dissociation of dt$\mu ^*$, $\Upsilon _{\mathrm {t}\mu }$, has never been predicted exactly and depends on the initial population and the subsequent Auger transitions among the resonance levels, we examine the $\lambda _c$ in the range of $0.1\le \Upsilon _{\mathrm {t}\mu }\le 0.9$. The EVM-SPM-FIF model almost reproduces the experimental values over a wide range of temperatures and $c_\mathrm {t}$. Note that the experimental data of $c_\mathrm {t}=0.4$ and $T\le 16$ K were obtained for the solid hydrogen target and might require additional theoretical treatment coupling to phonon interactions^28^.

It should be stressed that at the low $c_\mathrm {t}$ condition, the simple VM, $\eta _{\mathrm {dt}\mu }=1$ and $\lambda _{\mathrm {SPM}}=0$, significantly overestimates the cycle rate particularly at the small $c_\mathrm {t}$ conditions. In contrast to the VM, the VM-SPM model in which $\eta _{\mathrm {dt}\mu }=1$ and $\lambda _{\mathrm {SPM}}=5\times 10^{10}$ s$^{-1}$ provides a good agreement of calculated $\lambda _c$ with experiments at $c_\mathrm {t}=0.1$. Thus, the SPM processes play an indispensable role in description of d$\mu$ and t$\mu$ population drastically. Although the VM-SPM model does not reproduce the $\lambda _c$ at high $c_\mathrm {t}$ conditions, the EVM-SPM model gives closer results. As described below the Eq. (4), in previous studies of $\mu$CF kinetics model, a phenomenological factor $q_{1s}$, which represents the probability of a d$\mu ^*$ reaching d$\mu (1s)$, was introduced to explain the experimental observations^15,85^. In the present calculation, $q_{1s}$ is not explicitly used; instead, the SPM processes naturally alter the $q_{1s}$ tuning. As described in Ref.^73^, one of the reasons of this alternation is that the SPM processes open a way back from t$\mu (n=2)$ to d$\mu (1s)$ instead of the muon transfer reaction, d$\mu (n=2)$ + t $\rightarrow$ t$\mu (n=2)$ + d at the rate of 10$^{12}$ s$^{-1}$^84^. Another factor comes from dd$\mu ^*$ $\rightarrow$ d$\mu (1s)$ + d + $\gamma$, which prevents the $\mu$ in d$\mu (n=2)$ from transferring to the t, and enhances the probability that d$\mu (n=2)$ reaches d$\mu (1s)$. In turn, at the high $c_\mathrm {t}$ condition, the tt$\mu ^*$ formation/dissociation processes enhances the t$\mu (1s)$ fraction, which results in the small dependency on $\Upsilon _{\mathrm {t}\mu }$.

One can see the VM-SPM-FIF kinetics model, where $\eta _{\mathrm {dt}\mu }=1$ and $\lambda _{\mathrm {SPM}}=5\times 10^{10}$ s$^{-1}$ using $\Upsilon _{\mathrm {t}\mu }=0.5$, in the same figure. The scaling factor $\eta _{\mathrm {dt}\mu }$ does not change the $\lambda _c$ at small $c_\mathrm {t}$ conditions; however, $\eta _{\mathrm {dt}\mu }$ significantly contributes to $\lambda _c$ at high $c_\mathrm {t}$ and high *T* conditions. EVM-SPM assumes thermalization time scale of the hot muonic atoms to be 10$^7$ s$^{-1}$ at $\varphi =0.4$. The reproducibility of the experimental observations is improved by adding the FIF process to the EVM-SPM.

In order to investigate the IMF and FIF contributions to the total fusion yield $Y_\mathrm {f}$, we introduce partial fusion yields $Y_\mathrm {f}^{(i)}$ where *i* denotes IMF(dt$\mu$), IMF(dd$\mu$), IMF(tt$\mu$), and FIF. Figure 3b shows ratios $Y_\mathrm {f}^{(i)}/Y_\mathrm {f}$ for EVM-SPM-FIF and VM models at the condition of $\varphi =0.4$ and $T=900$ K. The similar trends can be seen at other temperatures. The IMF(dt$\mu$) has a major contribution to the $Y_\mathrm {f}$ in both models, and accounts for more than 95% of $Y_\mathrm {f}$ in the range of $0.1\le c_\mathrm {t}\le 0.8$ The contribution of IMF(dd$\mu$) strongly depends on the $c_\mathrm {t}$ and decreases as the $c_\mathrm {t}$ increases. The contribution of IMF(tt$\mu$), as expected, increases as the $c_\mathrm {t}$ increases. EVM-SPM-FIF model amplifies the contribution of IMF(dd$\mu$) and reduces that of IMF(tt$\mu$) from those of the VM. The FIF has a constant contribution to the fusion yield in the considered range, and has maximum contribution at $c_\mathrm {t}\sim 0.5$. This would be because the $c_\mathrm {t}\sim 0.5$ maximizes the probability to find hot t$\mu (1s)$-d and hot d$\mu (1s)$-t pairs.

**Figure 4:**
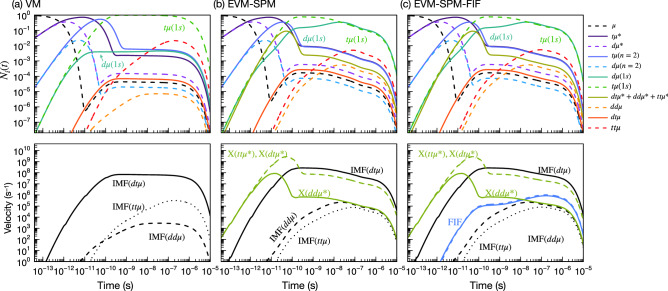
Upper panels: Time evolution of population of muonic atoms/molecules for (**a**) VM, (**b**) EVM-SPM, and (**c**) EVM-SPM-FIF kinetics models at $c_\mathrm {t}=0.5$, $\varphi =0.5$, and $T=800$ K. Lower panels: Velocities of nuclear fusion and X-ray emission events.

The upper panels of Fig. 4 display the time evolution of various muonic populations of VM/EVM-SPM/EVM-SPM-FIF kinetics model at $c_\mathrm {t}=0.5$, $\varphi =0.5$, and $T=800$ K. For brevity, we sum up some of the populations of the similar states, for example, t$\mu (1s)$ in $F=0,1$. It can be seen that the injected $\mu$ is replaced by an electron and forms t$\mu$/d$\mu$ atom which appears as a drop in the $\mu$ population around 10$^{-10}$ s. In accordance with the decrease of $\mu$, the muonic atom populations increase and then decrease due to molecular formation. Such a drastic change in the population boils down to a steady state in 10$^{-9}$ s. In this steady state, the VM calculation indicates that almost all of the $\mu$ exist as t$\mu (1s)$, and the population of d$\mu (1s)$ is more than two orders of magnitude smaller than that of t$\mu (1s)$. Since the $q_{1s}$ parameter is not include in the present models, the population of the tt$\mu$ is larger than the conventional model. Though the tt$\mu$ would be one factor to delay of the cycle because of its slow fusion rate, it gives broad neutron energy spectrum which can be a proof of the present models. In contrast to the VM, the EVM-SPM calculation results in the same amount of the populations, which could be because of the dissociation of dt$\mu ^*$. The similar time evolution can be found in EVM-SPM-FIF kinetics model.

In the lower panels of Fig. 4, the velocities of nuclear fusion and X-ray emission events are displayed. The largest contribution to the total fusion velocity is the IMF of dt$\mu$, and the second dominant effect comes from FIF processes, as we see in Fig. 4c. While the dt$\mu$ formation in VM(EVM) is only allowed for t$\mu$(1*s*) owing to the isotopic energy gap between d$\mu (1s)$ and t$\mu (1s)$, the FIF processes can be allowed not only for t$\mu (1s)$ + d collisions but also for d$\mu (1s)$ + t collisions, where the hot d$\mu (1s)$ are provided in the non-radiative dissociation of dt$\mu ^*$ *and* dd$\mu ^*$. The X-ray yields associated with the radiative dissociation of the resonant muonic molecules are constantly present during the $\mu$CF cycle.

For the test of the EVM-SPM-FIF kinetics model, one of the positive proofs of the experimental signals is the X-ray from resonant muonic molecules, dd$\mu ^*$, dt$\mu ^*$, and tt$\mu ^*$. As described above, these species emit characteristic X-rays whose energy spectrum ranging from 1.7 to 2.0 keV and can be, in principle, distinguished from the mono-energetic $2p\rightarrow 1s$ transition X-ray of muonic atoms, d$\mu$ and t$\mu$.

## Towards $\mu$CF in high temperature compressed gas targets

**Figure 5:**
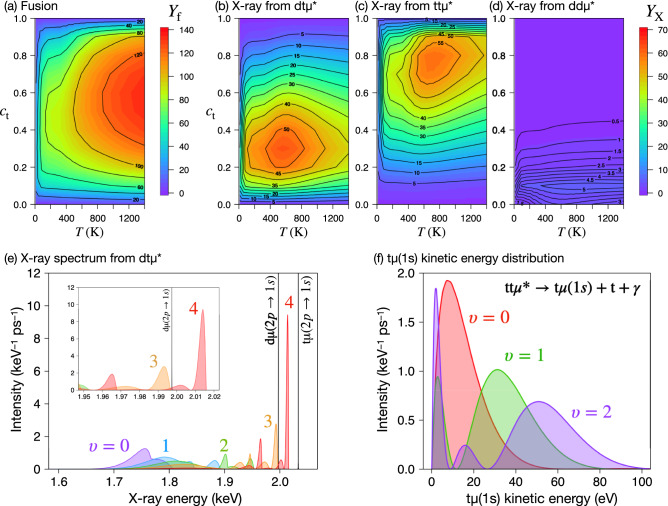
Fusion (**a**) and X-ray (**b**)–(**d**) yields as functions of tritium concentration and temperature under the $\varphi =1$ condition. (**e**) X-ray spectrum of $\mathrm {dt}\mu ^*$ in the $\upsilon$-th vibrational states (the colors indicate $0\le \upsilon \le 4$)^86^. The black narrow lines indicate $2p\rightarrow 1s$ X-rays of $\mathrm {d}\mu$ (1.997 keV) and $\mathrm {t}\mu$ (2.033 keV). The inset of (**e**) is a close-up view of the spectrum. (**f**) $\mathrm {t}\mu (1s)$ kinetic energy spectrum of radiative dissociation of $\mathrm {tt}\mu ^*$.

For future development of new $\mu$CF targets, it is worth to survey the $Y_\mathrm {f}$ of the EVM-SPM-FIF kinetics model as a function of temperature *T* and $c_\mathrm {t}$ together with the yields of X-rays $Y_\mathrm {X}$ from the resonant muonic molecules. The results obtained under the $\varphi =1$ condition are illustrated in Fig. 5. It can be seen in Fig. 5a that $Y_\mathrm {f}$ increases as the temperature increases and becomes maximum at $c_\mathrm {t}=0.4$–0.5.

From Fig. 5b–d, the contribution of the resonant muonic molecules is clearly indicated. At the $c_\mathrm {t}\sim 0.3$ condition, the dt$\mu ^*$ formation and dissociation are the dominant SPM processes. On the other hand, $c_\mathrm {t}\sim 0.75$ condition, the tt$\mu ^*$ formation and dissociation are the dominant SPM processes. It is seen that the dd$\mu ^*$ processes are almost suppressed. This is because the optimized formation rate, $\lambda _{\mathrm {dd}\mu ^*}=5\times 10^{10}$ s$^{-1}$ is much smaller than the muon transfer rate between the excited states, $10^{12}$ s$^{-1}$. The large contribution of tt$\mu ^*$ at high $c_\mathrm {t}$ condition explains the small dependency of $\lambda _c$ against $\Upsilon _{\mathrm {t}\mu }$ indicated in Fig. 3 because the tt$\mu ^*$ dissociation only enhances the t$\mu$(1*s*) populations. It should be also noted that the $Y_\mathrm {X}$ gradually decreases as the temperature increases. In the present model, the $2p\rightarrow 2s$ transition rates of muonic atoms are assumed to have temperature-dependency owing to the Lamb shift of these states (see the details in the subsection of kinetics model below). Accordingly, around the 400 K, the $2p\rightarrow 2s$ transition rates become comparable to the formation rate of resonant muonic molecules and then most of the 2*s* states of muonic atoms undergoes deexcitation at the higher temperature.

Figure 5e illustrates the superposition of X-ray spectra from several vibrational energy levels of $\mathrm {dt}\mu ^*$ ($0\le \upsilon \le 5$, $J=0$), for example. These spectra are taken from the recent calculation^86^ obtained by the three-body variational method, utilizing a Gaussian expansion method^87^ and a complex coordinate rotation method^79,88–90^. Since the $2p\rightarrow 1s$ X-rays from $\mathrm {d}\mu$ and $\mathrm {t}\mu$ are mono-energetic spectra while the X-ray radiation from $\mathrm {dt}\mu ^*$ has a broad and oscillating structure ranging from 1.7–2.03 keV depending on the vibrational state, the high energy-resolution X-ray detectors^91^ utilizing the superconducting transition should directly demonstrate the existence of dt$\mu ^*$ which is the key of the SPM processes. Figure 5e is based on a possible scenario where the resonant muonic molecule forms at the high vibrational state $\upsilon \sim 8$, and subsequently undergoes resonance-resonance transition by emitting an Auger electron, resulting in $\upsilon \lesssim 4$ states which dissociates emitting an X-ray photon. The required energy resolution can be seen in the inset of the Fig. 5e. The current energy resolution of the detector is $\sim 5$ eV (FWHM) at 6 keV^45^, which is promising to distinguish the nearest peak from the 2*p* 1*s* X-ray.

We calculate t$\mu (1s)$ kinetic energy spectrum after the radiative dissociation. The results are shown in Fig. 5f. Adding to the 20 eV t$\mu (1s)$ resulted from the isotopic muon transfer reaction (5) for $n=1$, most of the t$\mu$ atoms produced from the radiative dissociation of tt$\mu ^*$ have kinetic energy distribution of more than 20 eV. As described in the introduction, the difficulty in reasonably incorporating epi-thermal effects on $\mu$CF stems from the initial kinetic energy distribution of muonic atoms^67^. So far, the isotopic muon transfer reaction (5) and the Coulomb deexcitation have been considered to be a major source of the epi-thermal muonic atoms. As shown in the lower panels in Fig. 4, the dissociation X-ray yield $Y_\mathrm {X}$ accounts for the roughly half of the fusion yield $Y_\mathrm {f}$, which implies that the SPM process should have non-negligible contribution to the kinetic energy distribution of t$\mu$ and d$\mu$ atoms in the ground states.

Recently, a new $\mu$CF gas target utilizing the shock-wave compression (SWC) was proposed^92^. The SWC has different features from conventional adiabatic compression (AC) in which the temperature *T* and density $\varphi$ obey

8 $$\begin{aligned} \frac{\varphi }{\varphi _\mathrm {i}}=\left( \frac{T}{T_\mathrm {i}} \right) ^{\frac{1}{\gamma -1}}. \end{aligned}$$

$\gamma =1.4$ is the heat capacity ratio of the hydrogen. $T_\mathrm {i}$ and $\varphi _\mathrm {i}$ are the initial temperature and density of the gas, respectively. In the shock wave compression, however, $T/T_\mathrm {i}$ and $\varphi /\varphi _\mathrm {i}$ depends on the initial speed of the gas flow, $M_\mathrm {i}$ (Mach number; for supersonic flow, $1.2\le M_\mathrm {i}\le 5$) as

9 $$\begin{aligned} \frac{\varphi }{\varphi _\mathrm {i}}=\frac{(\gamma +1)M_\mathrm {i}^2}{(\gamma -1)M_\mathrm {i}^2+2}, \end{aligned}$$

and

10 $$\begin{aligned} \frac{T}{T_\mathrm {i}}=1+ \frac{2(\gamma -1)}{(\gamma +1)^2} \frac{\gamma M_\mathrm {i}^2+1}{M_\mathrm {i}^2} (M_\mathrm {i}^2-1). \end{aligned}$$

In the limits of $M_\mathrm {i}\rightarrow \infty$, for example,  $\varphi /\varphi _\mathrm {i}\rightarrow \mathrm {const.}$, and $T/T_\mathrm {i}\rightarrow \infty$.

**Figure 6:**
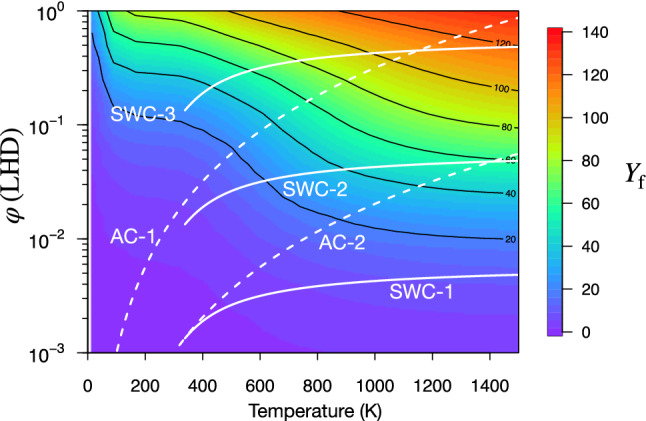
Fusion yields $Y_\mathrm {f}$ as functions of $\varphi$ and *T*. The white dashed lines denote $\varphi$-*T* relation of adiabatic compression (AC-1 assumes $T_\mathrm {i}=100$ K and $\varphi _\mathrm {i}=10^{-3}$; AC-2 assumes $T_\mathrm {i}=300$ K and $\varphi _\mathrm {i}=10^{-3}$). The white solid lines denote the relationship of shock wave compression (SWC-1 assumes 300 K and initial $\varphi _\mathrm {i}=10^{-3}$; SWC-2 assumes 300 K and initial $\varphi _\mathrm {i}=10^{-2}$; SWC-3 assumes 300 K and initial $\varphi _\mathrm {i}=10^{-1}$).

Figure 6 displays the $Y_\mathrm {f}$ as a function of temperature and the target density $\varphi$ under the condition of $c_\mathrm {t}=0.5$. As most of the rates of atomic processes except for radiative transitions depend on $\varphi$, the $Y_\mathrm {f}$ increases as $\varphi$ and *T* increase. White lines in Fig. 6 show possible thermodynamic processes for the future experiment (or experimental set up). As shown in Fig. 6, the high $Y_\mathrm {f}$ region could be achieved by adiabatic compression (white dashed lines) using $T_i=100$ K and $\varphi _\mathrm {i}=10^{-3}$ (approximately 1 atm). Three white solid lines indicate the different conditions of the SWC. We consider the gas jet of $M_\mathrm {i}$ at the $T_\mathrm {i}$ and $\varphi _\mathrm {i}$ initially. The *T* and $\varphi$ of the compressed gas depends on $M_\mathrm {i}$ that is a experimental tuning factor. SWC-1 assumes $T_\mathrm {i}=300$ K and $\varphi _\mathrm {i}=10^{-3}$, which can reach $Y_\mathrm {f}<20$. As seen in SWC-2 and SWC-3, increasing $\varphi _\mathrm {i}$, the highest $\varphi$ and $Y_\mathrm {f}$ increases.

In contrast to the AC, the SWC shows the limit of density. On the other hand, the temperature is easily tunable, which would be suitable for the high temperature $\mu$CF. While the AC is a *static* compression, the SWC is a *dynamical* compression that can be applied to realize the flowing gas target. Moreover, such a dynamic flow of the target will be utilized to extract energy and remove the helium atoms produced in $\mu$CF reaction.

## Conclusion

We proposed a new kinetics model of $\mu$CF and it showed a fairly good agreement with the experimental observations, without an unphysical tuning factor on the muonic atom population. The proposed kinetics model predicts that the cycle rate increase as an increase of temperature (800-1000 K). This kinetics model includes three roles of resonant muonic molecules, (i) changing isotopic population, (ii) producing epi-thermal muonic atoms, and (iii) inducing fusion in-flight. We also presented X-ray emission from the resonant muonic molecules that would provide a positive signal for verification of the $\mu$CF kinetics. We investigated the fusion yields in a wide range of temperatures $T\le 1500$ K and densities $10^{-3}\le \varphi \le 10^0$ LHD which can be prepared by the adiabatic or shock-wave compressions. The present results pave the way for future development of a $\mu$CF-based compact fusion reactor.

## Methods

### Numerical calculation

A full reaction scheme that reflects our actual calculations is illustrated in Fig. 2. We treat spin-flip reactions of muonic atoms in the ground state as a temperature-dependent processes.

Based on the full reaction scheme shown in Fig. 2, we have the following simultaneous ordinary differential equations. We denote the population of *i* (= $\mu$, d$\mu$(2*s*), dt$\mu$ and so on) as $N_i$.

11 $$\begin{aligned} \frac{ \mathrm {d}N_\mu }{ \mathrm {d}t }&= \left( -\lambda _0 -\lambda _a \varphi \right) N_\mu + \lambda _{\mathrm {IMF}(\mathrm {dt}\mu )} \left( 1-\tilde{\omega }_s\right) N_{\mathrm {dt}\mu } + \lambda _{\mathrm {IMF}(\mathrm {dd}\mu )} \left( 1-\omega _\mathrm {d}\Upsilon _{np}\right) N_{\mathrm {dd}\mu } \nonumber \\&\quad + \lambda _{\mathrm {IMF}(\mathrm {tt}\mu )} \left( 1-\omega _\mathrm {t}\right) N_{\mathrm {tt}\mu } + \lambda _\mathrm {FIF}c_\mathrm {d}\varphi \left( 1-\tilde{\omega }_s\right) N_{\mathrm {hot}\,\mathrm {t}\mu } + \lambda _\mathrm {FIF}c_\mathrm {t}\varphi \left( 1-\tilde{\omega }_s\right) N_{\mathrm {hot}\,\mathrm {d}\mu }, \end{aligned}$$

12 $$\begin{aligned} \frac{ \mathrm {d}N_{\mathrm {d}\mu ^*} }{ \mathrm {d}t }&= \left( -\lambda _0 -\lambda '_a \varphi \right) N_{\mathrm {d}\mu ^*} -\lambda '_{dt} c_\mathrm {t} \varphi N_{\mathrm {t}\mu ^*} + \lambda _a c_\mathrm {d} \varphi N_\mu \end{aligned}$$

13 $$\begin{aligned} \frac{ \mathrm {d}N_{\mathrm {t}\mu ^*} }{ \mathrm {d}t }&= \left( -\lambda _0 -\lambda '_a \varphi \right) N_{\mathrm {t}\mu ^*} +\lambda '_{dt} c_\mathrm {t} \varphi N_{\mathrm {d}\mu ^*} + \lambda _a c_\mathrm {t} \varphi N_\mu \end{aligned}$$

14 $$\begin{aligned} \frac{ \mathrm {d}N_{\mathrm {t}\mu (2s)} }{ \mathrm {d}t }&=\left\{ -\lambda _0 -\lambda _{2s\rightarrow 2p}(T) \varphi -\lambda _{\mathrm {SPM}} c_\mathrm {d}\varphi f_\mathrm {mol}(T) -\lambda _{\mathrm {SPM}} c_\mathrm {t}\varphi f_\mathrm {mol}(T) -\lambda _\mathrm {St} \right\} N_{\mathrm {t}\mu (2s)} \nonumber \\&\quad +\lambda '_a \varphi \Upsilon _s N_{\mathrm {t}\mu ^*} +\frac{1}{4} \lambda '_{dt} c_\mathrm {t} \varphi \left( N_{\mathrm {d}\mu (2s)} + N_{\mathrm {d}\mu (2p)} \right) + \lambda _{2p\rightarrow 2s}(T) \varphi N_{\mathrm {t}\mu (2p)} \end{aligned}$$

15 $$\begin{aligned} \frac{ \mathrm {d}N_{\mathrm {t}\mu (2p)} }{ \mathrm {d}t }&= \left\{ -\lambda _0 -\lambda _{2p\rightarrow 2s}(T) \varphi -\lambda _{\mathrm {SPM}} c_\mathrm {d}\varphi f_\mathrm {mol}(T) -\lambda _{\mathrm {SPM}} c_\mathrm {t}\varphi f_\mathrm {mol}(T) -\lambda _{2p\rightarrow 1s} \right\} N_{\mathrm {t}\mu (2p)} \nonumber \\&\quad +\lambda '_a \varphi (1-\Upsilon _s) N_{\mathrm {t}\mu ^*} +\frac{3}{4} \lambda '_{dt} c_\mathrm {t} \varphi \left( N_{\mathrm {d}\mu (2s)} + N_{\mathrm {d}\mu (2p)} \right) + \lambda _{2s\rightarrow 2p}(T) \varphi N_{\mathrm {t}\mu (2s)} \end{aligned}$$

16 $$\begin{aligned} \frac{ \mathrm {d}N_{\mathrm {d}\mu (2s)} }{ \mathrm {d}t }&= \left\{ -\lambda _0 -\lambda '_{2s\rightarrow 2p}(T) \varphi -\lambda _{\mathrm {SPM}} c_\mathrm {d}\varphi f_\mathrm {mol}(T) -\lambda _\mathrm {St} \varphi -\lambda '_{dt} c_\mathrm {t} \varphi \right\} N_{\mathrm {d}\mu (2s)} \nonumber \\&\quad +\lambda '_a \varphi \Upsilon _s N_{\mathrm {d}\mu ^*} + \lambda '_{2p\rightarrow 2s}(T) \varphi N_{\mathrm {d}\mu (2p)} \end{aligned}$$

17 $$\begin{aligned} \frac{ \mathrm {d}N_{\mathrm {d}\mu (2p)} }{ \mathrm {d}t }&= \left\{ -\lambda _0 -\lambda '_{2p\rightarrow 2s}(T) \varphi -\lambda _{\mathrm {SPM}} c_\mathrm {d}\varphi f_\mathrm {mol}(T) -\lambda _{2p\rightarrow 1s} -\lambda '_{dt} c_\mathrm {t} \varphi \right\} N_{\mathrm {d}\mu (2p)} \nonumber \\&\quad +\lambda '_a \varphi (1-\Upsilon _s) N_{\mathrm {d}\mu ^*} + \lambda '_{2s\rightarrow 2p}(T) \varphi N_{\mathrm {d}\mu (2s)} \end{aligned}$$

18 $$\begin{aligned} \frac{ \mathrm {d}N_{\mathrm {t}\mu (1s,F=0)} }{ \mathrm {d}t }&= \left\{ -\lambda _0 -\lambda _{\mathrm {dt}\mu (F=0)}(T) c_\mathrm {d} \varphi f_\mathrm {mol}(T) -\lambda _\mathrm {F=0\rightarrow 1}(T) c_\mathrm {t} \varphi -\lambda _{\mathrm {tt}\mu } c_\mathrm {t} \varphi f_\mathrm {mol}(T) \right\} N_{\mathrm {t}\mu (1s,F=0)} \nonumber \\&\quad +\frac{1}{4}\lambda _\mathrm {St} \varphi N_{\mathrm {t}\mu (2s)} +\frac{1}{4}\lambda _{2p\rightarrow 1s} N_{\mathrm {t}\mu (2p)} +\frac{1}{4}\lambda _{dt} c_\mathrm {t} \varphi \left( N_{\mathrm {d}\mu (1s,F=1/2)} + N_{\mathrm {d}\mu (1s,F=3/2)} \right) \nonumber \\&\quad +\frac{1}{4}\lambda _\mathrm {dis} \Upsilon _{\gamma } \left( N_{\mathrm {tt}\mu ^*} + \Upsilon _{\mathrm {t}\mu } N_{\mathrm {dt}\mu ^*} \right) +\frac{1}{4}\lambda _\mathrm {thr} \varphi N_{\mathrm {hot}\,\mathrm {t}\mu } +\lambda _\mathrm {F=1\rightarrow 0} c_\mathrm {t} \varphi N_{\mathrm {t}\mu (1s,F=1)} \end{aligned}$$

19 $$\begin{aligned} \frac{ \mathrm {d}N_{\mathrm {t}\mu (1s,F=1)} }{ \mathrm {d}t }&= \left\{ -\lambda _0 -\lambda _{\mathrm {dt}\mu (F=1)}(T) c_\mathrm {d} \varphi f_\mathrm {mol}(T) -\lambda _\mathrm {F=1\rightarrow 0} c_\mathrm {t} \varphi -\lambda _{\mathrm {tt}\mu } c_\mathrm {t} \varphi f_\mathrm {mol}(T) \right\} N_{\mathrm {t}\mu (1s,F=1)} \nonumber \\&\quad +\frac{3}{4}\lambda _\mathrm {St} \varphi N_{\mathrm {t}\mu (2s)} +\frac{3}{4}\lambda _{2p\rightarrow 1s} N_{\mathrm {t}\mu (2p)} +\frac{3}{4}\lambda _{dt} c_\mathrm {t} \varphi \left( N_{\mathrm {d}\mu (1s,F=1/2)} + N_{\mathrm {d}\mu (1s,F=3/2)} \right) \nonumber \\&\quad +\frac{3}{4}\lambda _\mathrm {dis} \Upsilon _{\gamma } \left( N_{\mathrm {tt}\mu ^*} + \Upsilon _{\mathrm {t}\mu } N_{\mathrm {dt}\mu ^*} \right) +\frac{3}{4}\lambda _\mathrm {thr} \varphi N_{\mathrm {hot}\,\mathrm {t}\mu } +\lambda _\mathrm {F=0\rightarrow 1}(T) c_\mathrm {t} \varphi N_{\mathrm {t}\mu (1s,F=0)} \end{aligned}$$

20 $$\begin{aligned} \frac{ \mathrm {d}N_{\mathrm {d}\mu (1s,F=1/2)} }{ \mathrm {d}t }&= \left\{ -\lambda _0 -\lambda _{\mathrm {dd}\mu (F=1/2)} c_\mathrm {d} \varphi f_\mathrm {mol}(T) -\lambda _{F=1/2\rightarrow 3/2}(T) c_\mathrm {t} \varphi - \lambda _{dt} c_\mathrm {t} \varphi \right\} N_{\mathrm {d}\mu (1s,F=1/2)} \nonumber \\&\quad +\frac{1}{3}\lambda _\mathrm {St} \varphi N_{\mathrm {d}\mu (2s)} +\frac{1}{3}\lambda _{2p\rightarrow 1s} N_{\mathrm {d}\mu (2p)} +\frac{1}{3}\lambda _\mathrm {dis} \Upsilon _{\gamma } \left( N_{\mathrm {dd}\mu ^*} + (1-\Upsilon _{\mathrm {t}\mu }) N_{\mathrm {dt}\mu ^*} \right) \nonumber \\&\quad +\frac{1}{3}\lambda _\mathrm {thr} \varphi N_{\mathrm {hot}\,\mathrm {d}\mu } +\lambda _{F=3/2\rightarrow 1/2} c_\mathrm {t} \varphi N_{\mathrm {d}\mu (1s,F=3/2)} \end{aligned}$$

21 $$\begin{aligned} \frac{ \mathrm {d}N_{\mathrm {d}\mu (1s,F=3/2)} }{ \mathrm {d}t }&= \left\{ -\lambda _0 -\lambda _{\mathrm {dd}\mu (F=3/2)} c_\mathrm {d} \varphi f_\mathrm {mol}(T) -\lambda _{F=3/2\rightarrow 1/2} c_\mathrm {t} \varphi - \lambda _{dt} c_\mathrm {t} \varphi \right\} N_{\mathrm {d}\mu (1s,F=3/2)} \nonumber \\&\quad +\frac{2}{3}\lambda _\mathrm {St} \varphi N_{\mathrm {d}\mu (2s)} +\frac{2}{3}\lambda _{2p\rightarrow 1s} N_{\mathrm {d}\mu (2p)} +\frac{2}{3}\lambda _\mathrm {dis} \Upsilon _{\gamma } \left( N_{\mathrm {dd}\mu ^*} + (1-\Upsilon _{\mathrm {t}\mu }) N_{\mathrm {dt}\mu ^*} \right) \nonumber \\&\quad +\frac{2}{3}\lambda _\mathrm {thr} \varphi N_{\mathrm {hot}\,\mathrm {d}\mu } +\lambda _{F=1/2\rightarrow 3/2}(T) c_\mathrm {t} \varphi N_{\mathrm {d}\mu (1s,F=1/2)} \end{aligned}$$

22 $$\begin{aligned} \frac{ \mathrm {d}N_{\mathrm {dt}\mu } }{ \mathrm {d}t }&= \left( -\lambda _0 -\lambda _{\mathrm {f}(\mathrm {dt}\mu )} \right) N_{\mathrm {dt}\mu } \nonumber \\&\quad +\lambda _{\mathrm {dt}\mu (F=0)}(T) c_\mathrm {d} \varphi f_\mathrm {mol}(T) N_{\mathrm {t}\mu (1s,F=0)} +\lambda _{\mathrm {dt}\mu (F=1)}(T) c_\mathrm {d} \varphi f_\mathrm {mol}(T) N_{\mathrm {t}\mu (1s,F=1)} \end{aligned}$$

23 $$\begin{aligned} \frac{ \mathrm {d}N_{\mathrm {dd}\mu } }{ \mathrm {d}t }&= \left( -\lambda _0 -\lambda _{\mathrm {f}(\mathrm {dd}\mu )} \right) N_{\mathrm {dd}\mu } \nonumber \\&\quad +\lambda _{\mathrm {dd}\mu (F=1/2)} c_\mathrm {d} \varphi f_\mathrm {mol}(T) N_{\mathrm {d}\mu (1s,F=1/2)} +\lambda _{\mathrm {dd}\mu (F=3/2)} c_\mathrm {d} \varphi f_\mathrm {mol}(T) N_{\mathrm {d}\mu (1s,F=3/2)} \end{aligned}$$

24 $$\begin{aligned} \frac{ \mathrm {d}N_{\mathrm {tt}\mu } }{ \mathrm {d}t }&= \left( -\lambda _0 -\lambda _{\mathrm {f}(\mathrm {tt}\mu )} \right) N_{\mathrm {tt}\mu } +\lambda _{\mathrm {tt}\mu } c_\mathrm {t} \varphi f_\mathrm {mol}(T) \left( N_{\mathrm {t}\mu (1s,F=0)} + N_{\mathrm {t}\mu (1s,F=1)} \right) \end{aligned}$$

25 $$\begin{aligned} \frac{ \mathrm {d}N_{\mathrm {dt}\mu ^*} }{ \mathrm {d}t }&= \left( -\lambda _0 -\lambda _{\mathrm {dis}} \right) N_{\mathrm {dt}\mu ^*} +\lambda _{\mathrm {SPM}} c_\mathrm {d} \varphi f_\mathrm {mol}(T) \left( N_{\mathrm {t}\mu (2s)} + N_{\mathrm {t}\mu (2p)} \right) \end{aligned}$$

26 $$\begin{aligned} \frac{ \mathrm {d}N_{\mathrm {dd}\mu ^*} }{ \mathrm {d}t }&= \left( -\lambda _0 -\lambda _{\mathrm {dis}} \right) N_{\mathrm {dd}\mu ^*} +\lambda _{\mathrm {SPM}} c_\mathrm {d} \varphi f_\mathrm {mol}(T) \left( N_{\mathrm {d}\mu (2s)} + N_{\mathrm {d}\mu (2p)} \right) \end{aligned}$$

27 $$\begin{aligned} \frac{ \mathrm {d}N_{\mathrm {tt}\mu ^*} }{ \mathrm {d}t }&= \left( -\lambda _0 -\lambda _{\mathrm {dis}} \right) N_{\mathrm {tt}\mu ^*} +\lambda _{\mathrm {SPM}} c_\mathrm {t} \varphi f_\mathrm {mol}(T) \left( N_{\mathrm {t}\mu (2s)} + N_{\mathrm {t}\mu (2p)} \right) \end{aligned}$$

28 $$\begin{aligned} \frac{ \mathrm {d}N_{\mathrm {hot}\,\mathrm {t}\mu (1s)} }{ \mathrm {d}t }&= \left( -\lambda _0 -\lambda _{\mathrm {thr}} -\lambda _\mathrm {FIF}c_\mathrm {d} \varphi \right) N_{\mathrm {hot}\,\mathrm {t}\mu (1s)} +\lambda _\mathrm {dis}\left( 1-\Upsilon _\gamma \right) \left( N_{\mathrm {dt}\mu ^*} \Upsilon '_\mathrm {t} + N_{\mathrm {tt}\mu ^*} \right) \end{aligned}$$

and

29 $$\begin{aligned} \frac{ \mathrm {d}N_{\mathrm {hot}\,\mathrm {d}\mu (1s)} }{ \mathrm {d}t } = \left( -\lambda _0 -\lambda _{\mathrm {thr}} -\lambda _\mathrm {FIF}c_\mathrm {t} \varphi \right) N_{\mathrm {hot}\,\mathrm {d}\mu (1s)} +\lambda _\mathrm {dis}\left( 1-\Upsilon _\gamma \right) \left( N_{\mathrm {dt}\mu ^*} (1-\Upsilon '_\mathrm {t})+ N_{\mathrm {dd}\mu ^*} \right) , \end{aligned}$$

where $\lambda _0=4.55\times 10^5$ s$^{-1}$^23^ is a $\mu$ decay rate, $\lambda _a=4\times 10^{12}$ s$^{-1}$^15^ is muonic atom ($\mathrm {d}\mu ^*$, $\mathrm {t}\mu ^*$) formation rate, $\lambda '_a=7\times 10^{10}$ s$^{-1}$^15^ is muonic atom cascade down rate, $\lambda _{\mathrm {IMF}(\mathrm {dt}\mu )}=1\times 10^{12}$ s$^{-1}$^15^ is intramolecular fusion rate of $\mathrm {dt}\mu$, $\lambda _{\mathrm {IMF}(\mathrm {dd}\mu )}=4\times 10^{8 }$ s$^{-1}$^15^ is intramolecular fusion rate of $\mathrm {dd}\mu$, $\lambda _{\mathrm {IMF}(\mathrm {tt}\mu )}=1.5\times 10^{7}$ s$^{-1}$^15^ is intramolecular fusion rate of $\mathrm {tt}\mu$, $\tilde{\omega }_s=\omega _s(1-R(T))$ is an effective $\alpha \mu$ sticking probability following the $\mathrm {dt}\mu$ IMF where $\omega _s=0.008$^15^ and *R*(*T*) is the reactivation fraction^24^, $\omega _\mathrm {d}=0.12$^15^ is the $\alpha \mu$ sticking probability following the $\mathrm {dd}\mu$ IMF and $\Upsilon _{n}=0.583$^15^ is $^3\mathrm {He}$ + n branching ratio, $\omega _\mathrm {t}=0.14$^15^ is the $\alpha \mu$ sticking probability following the $\mathrm {tt}\mu$ IMF, $\lambda '_{dt}\simeq 10^{12}$ s$^{-1}$^84^ is the rate of muon transfer reaction among the excited muonic atoms, namely, $\mathrm {d}\mu (n)$ + *t* $\rightarrow$ $\mathrm {t}\mu (n)$ + *d*, $\lambda _{dt}\simeq 10^{8}$ s$^{-1}$^15^ is muon transfer reaction rate at the ground state, $\mathrm {d}\mu (1s)$ + *t* $\rightarrow$ $\mathrm {t}\mu (1s)$ + *d*. $\lambda _{2s\rightarrow 2p}(T)$ and $\lambda _{2p\rightarrow 2s}$ are Stark mixing rates between 2*s* and 2*p* states. They are related each other as

30 $$\begin{aligned} \lambda _{2s\rightarrow 2p}(T)=3\exp \left( -\frac{\Delta E_\mathrm {Lamb}}{k_\mathrm {B}T}\right) \lambda _{2p\rightarrow 2s}, \end{aligned}$$

where $\Delta E_\mathrm {Lamb}\simeq 0.2$ eV denotes the Lamb shift of 2*s*-2*p* levels, $k_\mathrm {B}$ the Boltzmann constant, and $\lambda _{2p\rightarrow 2s}=10^{13}$ s$^{-1}$^73^, $\lambda _\mathrm {St}=10^9$ s$^{-1}$^73^ is the Stark mediated deexcitation rate of 2*s* muonic atoms. $10^{10}\le \lambda _{\mathrm {SPM}}\le 10^{12}$ s$^{-1}$ is the formation rate of resonant muonic molecules (in this work we use the same constant for $\mathrm {dd}\mu ^*$, $\mathrm {tt}\mu ^*$ and $\mathrm {dt}\mu ^*$ because of their high level density). $0\le f_\mathrm {mol}(T)\le 1$ is the fraction of target molecule calculated by the law of mass action,

31 $$\begin{aligned} \frac{n_\mathrm {at}}{n_{\mathrm {mol}}}=\frac{1}{n_\mathrm {at}} \left( \frac{\pi m_\mathrm {at}k_\mathrm {B}T}{h^2}\right) ^{\frac{3}{2}}\exp \left( -\frac{D}{k_\mathrm {B}T}\right) , \end{aligned}$$

where $n_{\mathrm {mol}}$ is the number density of molecules, $n_{\mathrm {at}}$ the number density of atoms, *D* the bond energy of hydrogen molecule, $m_\mathrm {at}$ the mass of the atom and *h* Planck constant. $\Upsilon _s$ is the probability of the excited muonic atom reaching the 2*s* state just after the cascade process (in this work we take 0.5 due to the parity conservation), $\lambda _{\mathrm {dt}\mu (F=0)}(T)$ and $\lambda _{\mathrm {dt}\mu (F=1)}(T)$ are $\mathrm {dt}\mu$ formation rates for $\mathrm {t}\mu (1s,F=0,1)$ (see main text), $\lambda _{\mathrm {tt}\mu }=2\times 10^6$ s$^{-1}$^15^ is the $\mathrm {tt}\mu$ formation rate, $\lambda _{\mathrm {dd}\mu (F=1/2)}=9\times 10^4$ s$^{-1}$^15^ and $\lambda _{\mathrm {dd}\mu (F=3/2)}=5\times 10^6$ s$^{-1}$ are $\mathrm {dd}\mu$ formation rates for $\mathrm {d}\mu (1s,F=1/2,3/2)$ (including non-resonant formation process), $\lambda _\mathrm {dis}=7\times 10^{10}$ s$^{-1}$ is the dissociation lifetime of resonant muonic molecules (estimated from^79^). $\Upsilon _{\gamma }\simeq 0.9$ is the branching ratio of radiative dissociation of the resonant muonic molecules, $0.1\le \Upsilon _{\mathrm {t}\mu }\le 0.9$ is the probability resulting in $\mathrm {t}\mu (1s)$ + *d* pair after the radiative dissociation of the resonant muonic molecules, $\Upsilon '_\mathrm {t}=0.1$^74^ is the probability resulting in $\mathrm {t}\mu (1s)$ + *d* pair after the non-radiative dissociation of the resonant muonic molecules, $\lambda _\mathrm {thr}$ is a typical thermalization time constant for the hot muonic atoms (we take 10$^7$ s$^{-1}$ in the present calculation based on classical interaction model and Monte Carlo calculations^68^). $\lambda _{F=3/2\rightarrow 1/2}$ and $\lambda _{F=1/2\rightarrow 3/2}(T)$^20^ are spin-flip reaction rate between $\mathrm {d}\mu (1s,F=3/2)$ and $\mathrm {d}\mu (1s,F=1/2)$ given by

32 $$\begin{aligned} \lambda _{F=1/2\rightarrow 3/2}(T)=2\exp \left( -\frac{\Delta E_{\mathrm {hfs}(\mathrm {d}\mu )}}{k_\mathrm {B}T}\right) \lambda _{F=3/2\rightarrow 1/2}, \end{aligned}$$

where $\Delta E_{\mathrm {hfs}(\mathrm {d}\mu )}=0.0485$ eV is the hyperfine splitting energy of $\mathrm {d}\mu (1s)$, and $\lambda _{3/2\rightarrow 1/2}=3\times 10^7$ s$^{-1}$. $\lambda _{F=1\rightarrow 0}$ and $\lambda _{F=0\rightarrow 1}(T)$ are spin-flip reaction rate between $\mathrm {t}\mu (1s,F=0)$ and $\mathrm {t}\mu (1s,F=1)$ given by

33 $$\begin{aligned} \lambda _{F=0\rightarrow 1}(T)=3\exp \left( -\frac{\Delta E_{\mathrm {hfs}(\mathrm {t}\mu )}}{k_\mathrm {B}(T+\Delta T)}\right) \lambda _{F=1\rightarrow 0}, \end{aligned}$$

where $\Delta E_{\mathrm {hfs}(\mathrm {t}\mu )}=0.24$ eV is the hyperfine splitting energy of $\mathrm {t}\mu (1s)$, and $\lambda _{F=1\rightarrow 0}=1.3\times 10^9$ s$^{-1}$. $\Delta T/k_\mathrm {B}\simeq 1.2$ eV is introduced to include the epi-thermal effect of spin-flip collisions.

Total sticking probability *W* can be given by

34 $$\begin{aligned} W&=\sum _F P_{\mathrm {d}\mu (1s,F)}\frac{\Upsilon _n c_\mathrm {d}\lambda _{\mathrm {d}\mathrm {d}\mu (F)}\omega _\mathrm {d}}{c_\mathrm {d}\lambda _{\mathrm {d}\mathrm {d}\mu (F)}+c_\mathrm {t}\lambda _\mathrm {dt}} +\sum _F P_{\mathrm {t}\mu (1s,F)}\frac{c_\mathrm {d}\lambda _{\mathrm {dt}\mu (F)}\tilde{\omega }_\mathrm {s} +c_\mathrm {t}\lambda _{\mathrm {tt}\mu }\omega _\mathrm {t}}{c_\mathrm {d}\lambda _{\mathrm {dt}\mu (F)}+c_\mathrm {t}\lambda _{\mathrm {tt}\mu }} \nonumber \\&+P_{\mathrm {hot}\,\mathrm {d}\mu (1s)} \frac{c_\mathrm {t}\lambda _\mathrm {FIF}\tilde{\omega }_\mathrm {s}}{c_\mathrm {t}\lambda _\mathrm {FIF}+\lambda _\mathrm {thr}} +P_{\mathrm {hot}\,\mathrm {t}\mu (1s)} \frac{c_\mathrm {d}\lambda _\mathrm {FIF}\tilde{\omega }_\mathrm {s}}{c_\mathrm {d}\lambda _\mathrm {FIF}+\lambda _\mathrm {thr}}, \end{aligned}$$

where $P_{\mathrm {d}\mu (1s,F)}$ and $P_{\mathrm {t}\mu (1s,F)}$ are *arrival* probabilities of $\mathrm {d}\mu (1s,F=1/2,3/2)$ and $\mathrm {t}\mu (1s,F=1,3)$, respectively. Similarly, $P_{\mathrm {hot}\,\mathrm {d}\mu (1s)}$ and $P_{\mathrm {hot}\,\mathrm {t}\mu (1s)}$ are the *arrival* probabilities of hot d$\mu$(1*s*) and t$\mu$(1*s*), respectively. These probabilities are defined by the relative value against the muonic atom formation probability.

### Future improvements of the kinetics model

Since the present kinetics model is based on the current few-body theories of muonic atoms/molecules. The theories do not cover the muonic atoms/molecules and surroundings because of the long-range Coulomb interactions. Thanks to the rapid progress of computer resources, the area where the few-body calculations can be handled is increasing, and the following improvements are expected in the model in the future.

First, the EVM is a simple implementation of epi-thermal muonic atom processes. It has been pointed out that the surrounding electron correction to the binding energy of dt$\mu$ (finite size effect)^93,94^ that affects the VM is insufficient^95,96^. Furthermore, the finite size effect should be treated much more rigorously in dt$\mu ^*$ than dt$\mu$ because the spatial size of dt$\mu ^*$ is much larger than that of dt$\mu$ and is close to the electron orbital. A rigorous four-body scattering calculation^97^ that can distinguish the final states of dt$\mu ^*$ radiative and non-radiative disociation will be required.

Second, there should be a discrepancy between the full-thermalization assumption and the realistic population of constituting species. The $\mu$ captured in atomic orbital cascades down to the lower levels converting the de-excitation energy to the kinetic energy of the muonic atom and the different kind of atomic/molecular processes from the fully thermalized processes would occur. So far such cascade processes were considered theoretically; however, as described above, not only the isotopic muon transfer at the ground states of the muonic atoms, but also the SPM processes (even by the radiative dissociation of resonant muonic molecules) induce epi-thermal muonic atoms. Theoretical implementation of the SPM contribution should be required.

Third, the rate of resonant muonic molecular formation which plays a crucial role in the $\mu$CF kinetics model with SPM should be tested experimentally. As shown in Fig. 5b–d, the yield of X-rays associated with the radiative dissociation is not small, and could be observable. The X-ray spectrum has characteristic structure depending on the vibrational states of dt$\mu ^*$ and dd$\mu ^*$ and can be distinguished from $2p\rightarrow 1s$ mono-energetic transition energy. Recently available X-ray detectors^43–45^ are promising tools.
